# The Hospital Officer's Bookshelf

**Published:** 1912-11-02

**Authors:** 


### THE HOSPITAL OFFICER'S BOOKSHELF.

With the Red Cross in the Franco-German War. By Henry Rundle, F.R.C.S. (London: Werner Laurie.)

The author of this booklet was one of a small band of St. Bartholomew's men who went to Germany on a Red Cross Mission during the Franco-Prussian War. His reminiscences of that campaign are now published by Mr. Rundle for the benefit of Portsmouth nurses, for all the profits from sales are to be given to the building fund of the Nurses' Home at the Royal Portsmouth Hospital. The passage of forty years and more has not dimmed Mr. Rundle's recollection of the battles and sieges in which he shared the lot of the German surgeons and soldiers. The siege of Strassburg, we read, cost the German army rather more than 900 lives; compared with the appalling carnage at Port Arthur this seems a very light list, but doubtless it was a busy time for the Red Cross people all the same. We wish Mr. Rundle all success in his effort to help the Nurses' Home, and many years of life in which to fight his battles over again.

Oscar Wilde: Art and Morality. By Stuart Mason. (Frank Palmer. 5s. net.)

This little book is a reprint containing a summary of the discussion excited by the publication of "Dorian Gray," a reprint of the principal reviews, including Walter Pater's, Wilde's letters to the newspapers concerning them, and various bibliographical matter. The psychological development of the story was the chief matter for abuse or discussion, and the psychologist of to-day can find in this summary of conflicting views almost as much matter for thought as in the story itself. If primarily directed to that literary public, for whom every scrap of Wilde's work has an almost apostolic value, the student of mental processes may not be sorry to place the book as a light commentary beside his theoretical volumes. That it is edited by Mr. Stuart Mason is sufficient for those who care for bibliography as a fine art to desire to possess it.

Studies in Clinical Medicine. By C. O. Hawthorne, M.D. (London: John Bale, Sons and Danielsson. 1912. Pp. 441. Price 6s. net.)

For some reason or another volumes of reprinted clinical lectures and essays have been less frequently issued of late years than was formerly the case. On the whole this is probably a tendency to be encouraged, for a collection of clinical lectures on very diverse topics is too ephemeral and too disjointed a product to be of any great value; while if they are all on the same or similar topics they are much less likely to be of use than a well-constructed monograph of the subject.

If anyone could keep alive the decaying custom to which allusion has been made, it would be Dr. Hawthorne. He has a great gift for setting forth succinctly and clearly the factors of any clinical problem with which he happens to be dealing, and for explaining the lines upon which the diagnostic differentiation is worked out. Several of the lectures and studies now reprinted originally appeared in The Hospital, and many of the others have seen the light in others of our contemporaries. One and all exhibit a power of consecutive reasoning and happy exposition which account for Dr. Hawthorne's reputation as a teacher. There is always a tendency in constructing clinical lectures to select for texts cases of unusual or rare disease, seldom to be encountered by any but the specialist. This temptation Dr. Hawthorne has been able to resist; he deals with common problems of diagnosis and treatment, preferring to deal with neglected aspects of them or unusual developments in individual cases. His collection of essays can be recommended strongly to any who have not time to wade through text-books and yet enjoy clinical instruction when they can get it good and in divided doses.

Suggestion and Psychotherapy. By G. W. Jacoby, M.D. (London: T. Fisher Unwin. 1912. Pp. 355. Price 7s. 6d. net.)

From America, land of Eddyism and a score of similar freaks and faddisms, comes as a corrective a really well balanced and sensible monograph dealing with the admitted facts concerning the influence of mind upon health and disentangling them from the masses of error which have so overgrown them. It is not going too far, states Dr. Jacoby, to say that the direct production of organic changes lies entirely beyond the possible effects of suggestion. Unfortunately the word "direct" rather detracts from the pronouncement, for it is an obvious loophole of retreat from a position which might prove untenable. To illustrate what we mean, it suffices to quote (from Dr. Jacoby) the experiments of Krafft-Ebing and Forel and the explanation thereof. These experimenters are said to have succeeded (in rare cases) in causing burns and blisters on the skin by suggesting that pieces of paper were glowing metal or mustard plaster respectively. Dr. Jacoby does not seem to be perfectly certain whether they really did obtain these results in the manner indicated; but assuming for the sake of argument that they did, he proceeds to explain them by arguing that the suggestion caused hyperæmia, and hyperæmia caused the lesions. We must confess this seems to us Jesuitical, and to be an unfair use of the loophole referred to above. What we should like to know is whether any such cases substantiated beyond the possibility of doubt really have been recorded. If they have, we would prefer to see Dr. Jacoby surrender his position openly rather than creep out of it by a quibble.

But to break a lance with this author over a small point is to run the risk of doing injustice unintentionally to a generally very sensible book. It contains an account of modern knowledge and recent theories which is as well adapted for the general public as for the medical profession. It avoids exaggeration and sensationalism, and it contains much that all who are interested in the care and cure of the sick ought to know. In other words, it is a book of none too common a stamp where this particular subject is concerned, and deserves to be recommended in consequence.

Text-Book of Hygiene for Teachers. By Robert A. Lyster, M.D., Ch.B., B.Sc., D.P.H. (London: W. B. Clive, University Tutorial Press, Ltd. 1912. Price 4s. 6d. net.)

An attempt to compress the essentials of hygiene, physiology, pathology, and pediatrics generally into a small volume must be well done or else it gives the impression that it is a mere compilation. We cannot honestly say that Dr. Lyster has achieved a success in his attempt to compress. Three-fourths of the information here given is known to every teacher who has any acquaintance with physiology. The book is, in our opinion, too elementary for teachers and too involved for pupil-teachers. There are many examples of irritating dogmatism in it—not the dogmatism of a teacher, but that of a special pleader who by assertion imagines he can entirely dispense with argument. For example, Dr. Lyster thinks that specialists *may* be necessary for a few special cases of eye, ear, or skin disease in school children, but that the school medical officer should do the inspection and treatment in the majority of schools. That is an opinion which we have always opposed, and which we think no one who has had practical experience of medical inspection of school children and of the treatment of diseases detected by such inspection will agree with. While the organisation of the general scheme of inspection ought to be supervised by the medical officer of health, the details and working of the scheme should be entrusted to men who have had practical experience of school children. The possession of the D.P.H. is no criterion, notwithstanding the views held by many education committees of the ability of the holder to diagnose and treat consumption in a school child.

We notice many curious methods of expression in this book which are examples of slipshod writing. Adenoids are stated to "feel soft, pulpy, and velvety like a bag of earthworms." We confess we have never felt a bag of earthworms, so we are not capable of judging the truth of this simile, but we do not see the necessity of giving such dubious information to teachers who should be the last persons to thrust their fingers behind the palate of children. There are many other sentences in the book which are fine examples of ambiguous or slovenly construction and which might well serve as suitable exercises for set corrections to a class. While such blemishes do undoubtedly mar Dr. Lyster's little book, his intention has obviously been to render assistance and help to the teacher who desires to know the elementary facts of the case for medical inspection in all its aspects. As such the book is welcome, but we venture to express two hopes—one that the next edition will be carefully sub-edited, preferably by a teacher who can correct the slovenly composition, and the other that the price of the book will be lowered to half-a-crown. Books of this nature should be cheap and lucidly written. These are indispensable characters, and we offer no apology for criticising rather severely points which seem to us to be of more importance in a popular treatise than in a scientific monograph—style and construction. The facts and information given by Dr. Lyster are accurate and the book is instructive, but its value will be enhanced by a better presentation of the facts and a clearer style of writing.

Deformities: Including Diseases of the Bones and Joints. By A. H. Tubby, M.S., F.R.C.S. 2 vols. Second Edition. (Macmillan and Co., Ltd. Price 45s. net.)

The first edition of this work was issued in 1896, and it says little for the general interest in orthopædics in this country that a second edition has not been required before. Yet the first was singularly handicapped by slovenly writing and a want of up-to-dateness that made a comparison between it and its Continental and American rivals a matter that eventuated solely in favour of the latter. Since its publication orthopædic surgery has advanced by leaps and bounds, and is to-day a recognised speciality which needs a specialist's treatment. And here it is well to state that a general surgeon's claim to be considered as an orthopædic surgeon would scarcely be admitted by many Continental enthusiasts who hold that an orthopædist should confine himself to orthopædics and not dabble in general, and especially in abdominal surgery. Those who recognise, as we do, that there is a wide difference between the art and what may be called the science of surgery, will not object to the claim on these grounds, since we hold that such claims must be tested by results, and that an all-round excellence in surgery is not a drawback but an advantage to the orthopædist.

In this new edition Mr. Tubby has shown that he has the ability and the knowledge to be a teacher in the subject to which he has specially applied himself. The volumes have been rewritten, and excellent use has been made of much of the recent literature. If the more recent authorities have not been laid under such large contribution as the student, who desires such a text-book to be almost encyclopædic in its references to the literature, may wish, the reader must yet admit that many and, on the whole, very excellent references are given, although little mention is made of Russian, Italian, and Dutch authorities. The theoretical part of the book is almost wholly taken from the best authorities, and Mr. Tubby gives, in nearly every case, both sides of the question when there is material difference of opinion. This enhances the value of the work, and makes it really the best exposition of the subject with which it treats that has yet been published in English. To instance specific cases and to deal *in extenso* with the many important points raised in the book would take up more space than we can give to it here. Nor perhaps would such criticism be fair, since it concerns not primarily the author's views but those of pioneers whose opinions are well known to experts. To the practitioner Mr. Tubby's book is valuable because it gives a good account of treatment. Take the question of lateral curvature of the spine. We do not agree with Mr. Tubby regarding the over-mastering influence of school furniture in the etiology of this condition: such influence, in our opinion, has been much over-estimated, and certainly the orthopædist who only sees the patients, and not the mass of school children, is not the proper person to give an opinion unless he is at the same time a school doctor. We think, too, that the author lays too little stress on eye defects (producing the so-called Landolt's deformities). But these are side issues. When we come to the question of treatment we find that Mr. Tubby gives a very fair, correct, and judicious account of all the modern methods which should prove of very great value to the practitioner. Perhaps on one point only we should quarrel with him—his footnote on page 554, which shows that he has not had any experience of the Klapp method of treating scoliosis. It is not correct to call this a "creeping exercise" only, and it is equally incorrect to state that it leads to "a high, ungainly position of the shoulders." Nor is it a recent method. It has been used for the last ten years and has proved a singularly good adjunct to the ordinary methods. In his description of the methods for the treatment of flat-foot, the author does not describe the simplest way of obtaining casts and moulds of the foot, nor does he detail the technique of celluloid work, being apparently unfamiliar with it. Operative treatment is rightly given a less prominent place, where it is secondary to manipulative methods, and everywhere Mr. Tubby has arranged his notes as to treatment in a very lucid and clear manner. The illustrations given greatly add to the value of the work, particularly in the second volume, in which the important subject of nerve lesions is dealt with. The treatment of such lesions, although strictly a matter for the specialist, has often to be undertaken by the general practitioner, who will find in this work a very valuable help. The publishers have done their share of the book admirably. It is well printed and bound, and there are remarkably few printers' errors. But it would have been improved by stricter sub-editing. We confess we do not really know what such a word as "traumatism" means: it is easy to understand what the author intends to imply when he uses it, but the term is not correct and should be deleted.

